# Capulet and Slingshot share overlapping functions during *Drosophila* eye morphogenesis

**DOI:** 10.1186/1423-0127-19-46

**Published:** 2012-04-30

**Authors:** Chiao-Ming Lin, Pei-Yi Lin, Yu-Chiao Li, Jui-Chou Hsu

**Affiliations:** 1 Institute of Molecular Medicine, Department of Life Science, National Tsing Hua University, Hsinchu, Taiwan, 30034, Republic of China; 2 Department of Biological Science and Technology, National Chiao Tung University, Hsinchu, Taiwan, 30034, Republic of China

**Keywords:** Capulet, Slingshot, Twinstar, F-actin, Eye development

## Abstract

**Background:**

CAP/Capulet (Capt), Slingshot (Ssh) and Cofilin/Twinstar (Tsr) are actin-binding proteins that restrict actin polymerization. Previously, it was shown that low resolution analyses of loss-of-function mutations in *capt, ssh* and *tsr* all show ectopic F-actin accumulation in various *Drosophila* tissues. In contrast, *RNAi* depletion of *capt, tsr* and *ssh* in *Drosophila* S2 cells all affect actin-based lamella formation differently. Whether loss of these three related genes might cause the same effect in the same tissue remains unclear.

****Methods**:**

Loss-of-function mutant clones were generated using the MARCM or EGUF system whereas overexpression clones were generated using the Flip-out system. Immunostaining were then performed in eye imaginal discs with clones. FRAP was performed in cultured eye discs.

**Results:**

Here, we compared their loss-of-function phenotype at single-cell resolution, using a sheet of epithelial cells in the *Drosophila* eye imaginal disc as a model system. Surprisingly, we found that *capt* and *ssh*, but not *tsr*, mutant cells within and posterior to the morphogenetic furrow (MF) shared similar phenotypes. The *capt*/*ssh* mutant cells possessed: (1) hexagonal cell packing with discontinuous adherens junctions; and (2) largely complementary accumulation of excessive phosphorylated myosin light chain (p-MLC) and F-actin rings at the apical cortex. We further showed that the *capt/ssh* mutant phenotypes depended on the inactivation of protein kinase A (PKA) and activation of Rho.

**Conclusions:**

Although Capt, Ssh and Tsr were reported to negatively regulate actin polymerization, we found that Capt and Ssh, but not Tsr, share overlapping functions during eye morphogenesis.

## Background

Remodeling of the actin cytoskeleton is controlled by various groups of actin-binding proteins that function at different steps to promote dynamic F-actin assembly and disassembly. Cyclase-associated protein (CAP) homologs have been suggested to act as actin monomer sequestering proteins through their C-terminal actin-binding domains to suppress the spontaneous polymerization of actin [1,2]. Cofilin, an actin depolymerization factor, severs and depolymerizes older F-actin from the pointed end of the filament [3]. Cofilin is inactivated by phosphorylation of an N-terminal serine by LIM kinase [4] and activated by removal of the phosphate by Ssh, a cofilin phosphatase [5].

*capt* and *tsr* encode the *Drosophila* CAP and cofilin orthologues, Capulet (Capt) and Twinstar (Tsr), respectively. Loss-of-function mutations in *capt, ssh* and *tsr* all cause increased ectopic accumulation of F-actin in various *Drosophila* tissues that leads to defects in epithelial morphogenesis [5-9]. For example, it has been shown [7] that Capt, by affecting actin polymerization, alters cell shape and therefore affects the distribution of Hh. However, *RNAi* depletion of *capt, tsr*, and *ssh* in *Drosophila* S2 cells affects actin-based lamella formation differently [10].

*Drosophila* retinal differentiation starts in third instar larvae when a moving MF sweeps across the developing eye disc in a posterior-to-anterior direction. Cells anterior to the MF proliferate and have a large apical surface whereas cells within the MF undergo apical constriction. Moreover, cells posterior to the MF differentiate and form ommatidial clusters that secrete Hh proteins [11,12], whereas the remaining interommatidial cells (ICs) relax and regain a large apical surface. The anteriorly-diffused Hh activates the expression of target genes such as *dpp* and *atonal* in a strip of cells immediately ahead of the MF [13]. The transient activation of the Hh pathway in these cells converts Ci from a repressor (Ci75) to an activator (Ci155). Hh-mediated apical constriction in the MF requires RhoA/Rho kinase and other unidentified kinases that act through both myosin II and Diaphanous to respectively cause the enrichment of activated myosin II and F-actin in the apical cortex of these cells [9,14]. Rho kinase activates myosin II via phosphorylation of Ser19 of the MLC.

In this report, we compared the loss-of-function phenotypes of *capt, tsr*, and *ssh* using the sheet of epithelial cells in the *Drosophila* eye imaginal disc as a model system. We demonstrated that *capt* and *ssh*, but not *tsr*, mutants within and posterior to the MF similarly showed: (1) largely complementary accumulation of excessive F-actin and p-MLC, and (2) hexagonal cell packing with discontinuous AJs in the eye epithelial cells. We also found that these phenotypes depended on the inactivation of PKA and activation of Rho. Thus, Capt and Ssh have common functions in regulating actin depolymerization during eye morphogenesis.

## Methods

### Drosophila genetics

The following stocks were used: *capt*^*E636*^[7]*, ssh*^*1-63*^[5], *tsr*^*N96A*^[15], UAS-R*[16], *twf*^*110*^[17], *UAS-Rho*^*N19*^[18], *ubi-DE-cad-GFP*[19].

Loss-of-function mosaic clones were generated using the MARCM system [20]. Entirely *capt*^*E636*^ or *ssh*^*1-63*^ mutant eyes were generated using the EGUF (*eyeless*-Gal4 UAS-FLP) system of recombination [21]. Flip-out clones were generated by *P[act5C > y+ > GAL4] P[UAS-GFP.S65T]/CyO*[22].

### Histochemistry and confocal quantification

For immunostaining, third instar larval eye imaginal discs were dissected and fixed in 4% paraformaldehyde. Antibodies used were: rat anti-DE-cad (DCAD2; 1:50; Hybridoma Bank), mouse anti-Arm (N2-7A1; 1:40; Hybridoma Bank), rabbit anti-pSer19-MLC (1:10; Cell Signaling Technology), rat anti-Ci155 (2A1; 1:1; Hybridoma Bank), rabbit anti-Egfr (1:50; [23]), Alexa 594-phalloidin (1:200; Invitrogen), and Cy3- and Cy5-conjugated secondary IgGs (Jackson Immuno Research Laboratories).

Images were acquired using a 63× NA1.4 Oil Plan-Apochromat objective lens on a confocal microscope (LSM510, Carl Zeiss). The fluorescence intensity and apical surface area were quantified using Zeiss LSM software. The percentage of cells with x-sided polygons was quantified manually by measuring the number of neighboring cells. For fluorescence recovery after photobleaching (FRAP), third instar larval eye discs with *ubi-DE-cad-GFP* were dissected and placed in a drop of serum-free M3 medium at room temperature. A small area (1500 nm^2^) was photobleached. Fluorescence intensity in the bleached region or tricellular junction was measured at each time point using Zeiss LSM software.

## Results

### *Capt* mutant phenotype behind and within the MF

**Figure 1:**
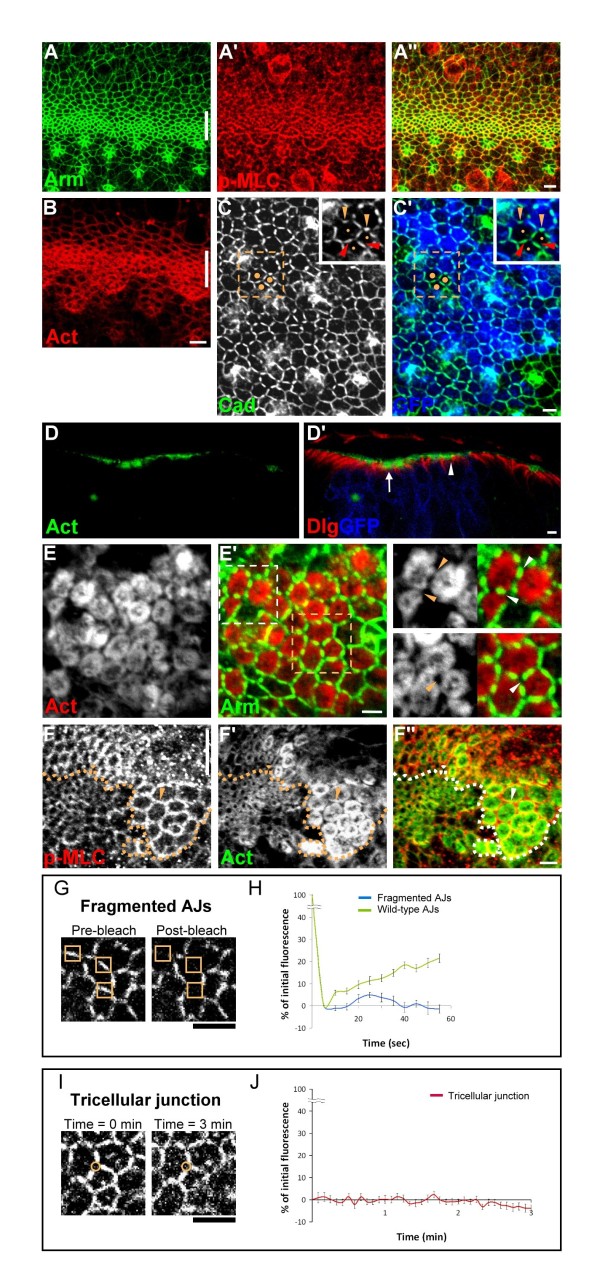
***capt*mutant phenotype behind the MF.** (A,B) Confocal images of wild-type third instar larval eye imaginal discs stained for Arm (green in A), p-MLC (red in A) and actin (red in B). Lines indicate the position of the MF, and anterior is to the top. Bars, 2 μm. (C) *capt*^*E636*^ mutant MARCM clones (GFP, blue) posterior to the MF labeled for DE-cad (green). Inset: High magnification image of three wild-type ICs (indicated by dots, without GFP) shows fragmented AJs (orange arrowheads) at the interface between wild-type (dots) and *capt* mutant ICs (no dots) as well as fragmented AJs (red arrowheads) at the interface between wild-type ICs (dots). (D) Sagittal section of *capt*^*E636*^ mutant MARCM clones (GFP, blue) stained for actin (green) and Dlg (red). The MF and the area posterior to the MF are indicated as an arrow and an arrowhead, respectively. (E) Entirely *capt*^*E636*^ mutant eyes (posterior to the MF) stained for actin (red) and Arm (green). Upper inset: *capt*^*E636*^ mutant cells under high magnification (indicated by white dashed box in E′) shows AJs formed at regions with proximate F-actin rings (arrowheads). Lower inset: *capt*^*E636*^ mutant cells under high magnification (indicated by orange dashed box in E′) shows AJs failed to form at the tricellular junction where F-actin was absent (arrowhead). Anterior is to the top. Bars, 2 μm. (F) *capt*^*E636*^ mutant MARCM clones posterior to the MF (arrowhead) labeled for p-MLC (red) and actin (green). Dashed lines indicate clone borders. Lines indicate the position of the MF, and anterior is to the top. Bars, 2 μm. (G-J) Fragmented AJs were relatively stable and did not diffuse laterally towards the tricellular junction. (G,I) Images of *ubi-DE-cad-GFP* in cultured entirely *capt*^*E636*^ mutant eye discs. Boxes in G indicate an area (1500 nm^2^) of the fragmented AJs before and after bleach, and circles in I indicate an area (350 nm^2^) of a tricellular junction at zero and 3 min time points. (H) Averaged FRAP recovery curves for fragmented AJs (blue) and wild-type AJs (green). (J) Fluorescence intensity curves for the tricellular junction. Each data point represents the fluorescence intensity measured at each time point divided by the initial fluorescence of the adjacent fragmented AJs. Bars, 2 μm.

It was previously shown that loss of *capt, ssh* and *tsr* in the *Drosophila* eye epithelia, at low resolution, all caused similar accumulation of F-actin and enlarged apical area [5,7,9]. To determine whether the accumulation of excess F-actin caused by loss of *capt, tsr* and *ssh* might differentially affect epithelial morphogenesis, we compared the phenotypes of the loss-of-function null alleles (*capt*^*E636*^*, tsr*^*N96A*^ and *ssh*^*1-63*^) at high magnification using the MARCM system (marked by the presence of CD8-GFP expression) in third instar eye imaginal discs [20] or by generating eye discs entirely mutant for *capt/ssh*[21]. Cells in the MF have a small apical surface area (Figure 1A). Consistent with the purse-string model for apical constriction, MF cells also accumulated activated myosin II (revealed by p-MLC antibody staining, Figure 1A′) and F-actin (revealed by phalloidin staining, Figure 1B) that colocalize with circumferential AJs (revealed by Armadillo (Arm) antibody staining, Figure 1A). Behind the MF, the photoreceptor clusters that formed remained apically constricted whereas the ICs, with low levels of Arm, p-MLC and F-actin, relaxed and adopted irregular cell shapes (Figure 1A, B). As previously reported [7], *capt*^*E636*^ mutant ICs often had enlarged surface areas (Additional file 1: Figure S1). However, we additionally found that these cells, possibly due to minimization of surface free energy, often adopted a hexagonal cell shape (62 ± 2%, *n* = 450 cells in entirely *capt*^*E636*^mutant eye disc *vs.* 24 ± 2%, *n* = 420 cells in wild-type eye discs, Additional file 1: Figure S2) and possessed discontinuous AJs with a higher density of DE-cadherin (DE-cad) (Figure 1C). These fragmented AJs were mainly localized to the center of each side of the hexagon, with AJs missing at the tricellular junctions (Figure 1C). Occasionally, more than one smaller fragmented AJ could be seen on the side of a hexagon (inset in Figure 1E′). Similar phenotypes were also detected with other AJ markers such as Echinoid and α-catenin (data not shown), indicating defects in the establishment/maintenance of circumferential AJs. Importantly, the discontinuous AJs were detected not only along the interface between *capt*^*E636*^ and *capt*^*E636*^ mutant cells but also between wild-type and *capt*^*E636*^ mutant cells (orange arrowheads in inset of Figure 1C). Thus, the discontinuous AJs formed on one side of a *capt*^*E636*^-wild-type interface, possibly via homophilic interaction of DE-cad, affected the distribution of AJs on the adjacent wild-type cells. Moreover, the discontinuous AJs could also be non-autonomously detected along the interface between wild-type cells when they were surrounded by the *capt*^*E636*^ mutant cells (red arrowheads in inset of Figure 1C, see Discussion).

Next, we determined whether loss of Capt in the ICs behind the MF affected the distribution of the actomyosin network. Unlike wild-type ICs where both p-MLC and F-actin were barely detectable, we observed strong apical accumulation of F-actin into a ring-like structure in sub-cortical regions of the *capt*^*E636*^ mutant ICs (Figure 1D, E). Of note, the fragmented AJs formed in regions where F-actin rings from two adjacent *capt*^*E636*^ mutant ICs came close together (arrowheads in upper inset of Figure 1E′) whereas AJs failed to form at the tricellular junctions where F-actin was absent (arrowhead in lower inset of Figure 1 E′). Surprisingly, we also observed dramatic accumulation of p-MLC at the apical cortex of *capt*^*E636*^ mutant ICs (arrowhead in Figure 1F), with the levels of p-MLC accumulation much higher than wild-type ICs (315 ± 12%, *n* = 21 cells). Importantly, this strong p-MLC staining outlined the cell morphology and partially overlapped with the F-actin ring (Figure 1F). Thus, fragmented AJs assemble at regions where cadherin/catenin, F-actin and myosin II overlap but do not assemble at the tricellular junction where F-actin is missing.

Next, we determined the dynamics of DE-Cad within the fragmented AJs by analyzing the distribution of *ubi-DE-cad-GFP* in cultured entirely *capt*^*E636*^ mutant eye discs. As expected, DE-Cad-GFP localized to the middle of each side of the hexagon but was missing at the tricellular junction (Figure 1G). In tests of fluorescence recovery after photobleaching (FRAP) in an area (1500-nm^2^) of the fragmented AJs in *capt*^*E636*^ mutant cells, we found no significant recovery of fluorescence signal for over 55 s (Figure 1H). This is in contrast to the control, which showed faster recovery of DE-Cad-GFP fluorescence when we performed FRAP in the wild-type AJs (1500-nm^2^) of *ubi-DE-cad-GFP* discs (Figure 1H). Moreover, the fluorescence intensity in the tricellular junction of *capt*^*E636*^ mutant cells was very low, and we found that the fluorescence signal in a small area (350-nm^2^) of a tricellular junction did not significantly change for up to 3 minutes (Figure 1I, J). Together, our results suggest that the fragmented AJ was relatively stable and did not diffuse laterally towards the tricellular junction.

**Figure 2:**
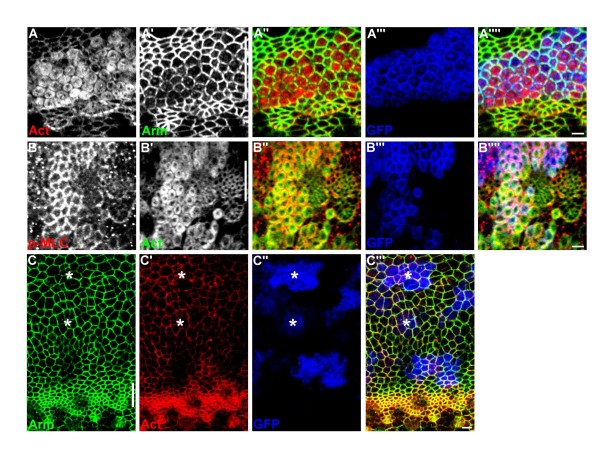
***capt*mutant phenotype was detected within the MF, but not anterior to the MF.** (A,B) *capt*^*E636*^ mutant MARCM clones (GFP, blue) within the MF show partial overlap between the sub-cortically localized F-actin ring (red in A) and AJs (Arm, green in A) as well as between the sub-cortically localized F-actin ring (green in B) and cortical p-MLC (red in B). (C) *capt*^*E636*^ mutant MARCM clones (GFP, blue) anterior to the MF (asterisks) labeled for Arm (green) and F-actin (red). In all images, lines indicate the position of the MF, and anterior is to the top. Bars, 2 μm.

The *capt*^*E636*^ mutant phenotype in ICs described above could also be detected in *capt*^*E636*^ mutant cells within the MF (Figure 2A,B), but, interestingly, not ahead of the MF (Figure 2C, and other data not shown). Due to the smaller apical surface of MF cells, the features of hexagonal cell shape and discontinuous AJs were less evident in *capt*^*E636*^ mutant MF cells (Figure 2A′). However, the *capt*^*E636*^ mutant MF cells had significantly enlarged apical area compared to endogenous MF cells (Figure 2A,B, Additional file 1: Figure S1) [7,9]. Unlike the endogenous MF cells where p-MLC and F-actin strongly colocalize with circumferential AJs, we again observed partial overlap between the sub-cortically localized F-actin ring and AJs (Figure 2A) as well as between the sub-cortically localized F-actin ring and cortical p-MLC (Figure 2B) in *capt*^*E636*^ mutant MF cells. Of note, the levels of p-MLC accumulated in the *capt*^*E636*^ mutant MF cells were much higher than in the endogenous MF cells (216 ± 11%, *n* = 34 cells, Figure 2B). Mature AJs contain continuous E-cadherin that links to a circumferential belt of actin filaments that associate with the bipolar myosin II minifilaments to form the contractile actomyosin network [24]. We speculate that the formation of discontinuous DE-Cad/AJ might prevent these *capt* mutant MF cells from full constriction that in turn caused the cell enlargement and the distortion of the MF (see Discussion). Together, our results demonstrate that *capt* plays an important role in cells within and posterior to the MF, and loss of Capt not only affects F-actin polymerization, as reported, but also MLC phosphorylation/activation, the continuity of AJs, and changes cell shape.

### *Capt* and *ssh* mutants share a similar phenotype

**Figure 3:**
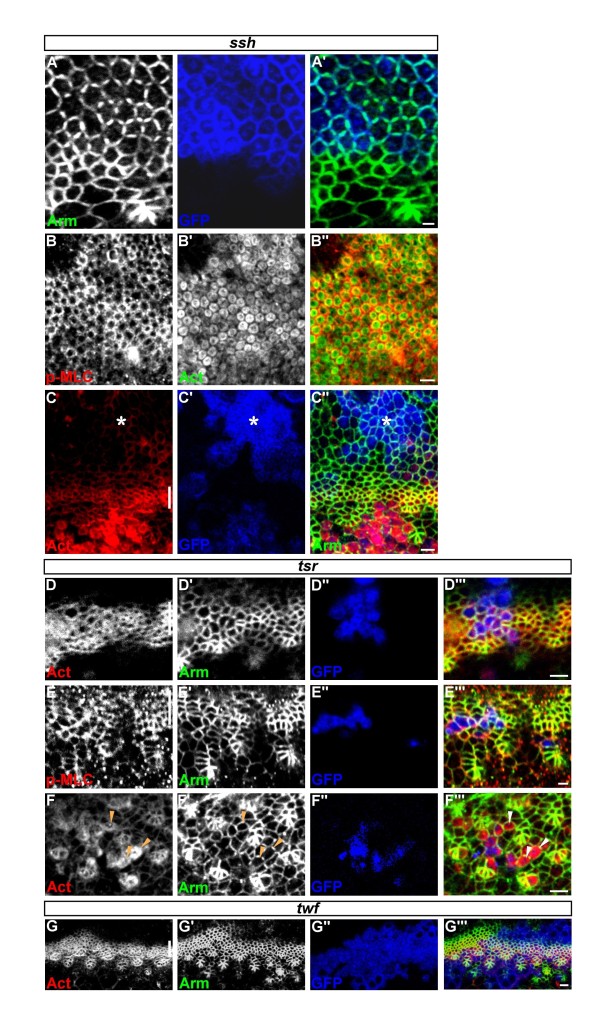
***ssh*, but not*tsr*, share a similar phenotype to*capt***. (A-C) *ssh*^*1-63*^ mutant phenotype. (A) *ssh*^*1-63*^ mutant MARCM clones (GFP, blue) labeled for Arm (green). (B) Large *ssh*^*1-63*^ mutant MARCM clones labeled for p-MLC (red) and actin (green). (C) Strong accumulation of F-actin (red) was detected only in *ssh*^*1-63*^ mutant MARCM clones (GFP, blue) posterior but not anterior (asterisk) to the MF. (D-F) *tsr*^*N96A*^mutant phenotype. (D,E) *tsr*^*N96A*^ mutant MARCM clones (GFP, blue) within the MF labeled for F-actin (red in D), p-MLC (red in E) and Arm (green). (F) *tsr*^*N96A*^ mutant MARCM clones (GFP, blue) posterior to the MF labeled for F-actin (red) and Arm (green). Arrowheads indicate *tsr*^*N96A*^ mutant ICs with elevated F-actin. (G) No excessive F-actin (red) accumulation, hexagonal cell shape or discontinuous AJs (Arm, green) were detected in *twf*^*110*^ mutant MARCM clones (GFP, blue) within and posterior to the MF. In all images, lines indicate the position of the MF, and anterior is to the top. Bars, 2 μm.

*ssh* encodes Slingshot, a cofilin phosphatase, and *ssh* mutant clones in the eye showed a strong accumulation of F-actin and an enlarged apical surface [5,9]. Interestingly, we found that *ssh*^*1-63*^ mutant clones within and posterior to the MF exhibited similar phenotypes to *capt*^*E636*^ mutant clones. These included (1) adoption of a hexagonal cell shape and discontinuous AJs (Figure 3A) and (2) strong accumulation of p-MLC at the apical cortex which partially overlapped the ectopic F-actin ring in the sub-cortical region (Figure 3B). Moreover, similar to *capt*, these phenotypes could not be detected in *ssh*^*1-63*^ mutant clones located anterior to the MF (Figure 3C).

Ssh positively regulates cofilin/Tsr activity to promote F-actin severing and depolymerization [5]. We thus looked next at clones mutant for Tsr, using the *tsr*^*N96A*^ mutation. Compared to the endogenous MF cells, *tsr*^*N96A*^ mutant cells in the MF showed enlarged apical surface as reported [9]. However, unlike *cap*^*E636*^ and *ssh*^*1-63*^ MF cells, *tsr*^*N96A*^ MF cells contained largely normal levels and distribution of F-actin and p-MLC that overlapped with AJs (Figure 3D,E). Moreover, these cells did not adopt a hexagonal cell shape (Figure 3D,E). *tsr*^*N96A*^ mutant cells posterior to the MF did show strong accumulation of cortical F-actin (Figure 3F) and, possibly via apical constriction, often led to groove formation when the clones were large (data not shown). Thus, although *capt, ssh* and *tsr* all affected the apical surface, *capt* and *ssh* differed from *tsr* in their effects on F-actin polymerization, MLC phosphorylation and cell-shape change within the MF.

Twinfilin (Twf), similar to ADF/cofilin, sequesters actin monomers and negatively regulates F-actin formation [25]. However, we did not detect excessive F-actin accumulation, hexagonal cell shape, or discontinuous AJs when *twf*^*110*^ null allele mutant clones were generated using MARCM within and posterior to the MF (Figure 3G and data not shown). Together, our results suggest that the effects in eye epithelial cells are *capt/ssh*-specific.

### *Capt* mutant phenotype depends on the inactivation of PKA

**Figure 4:**
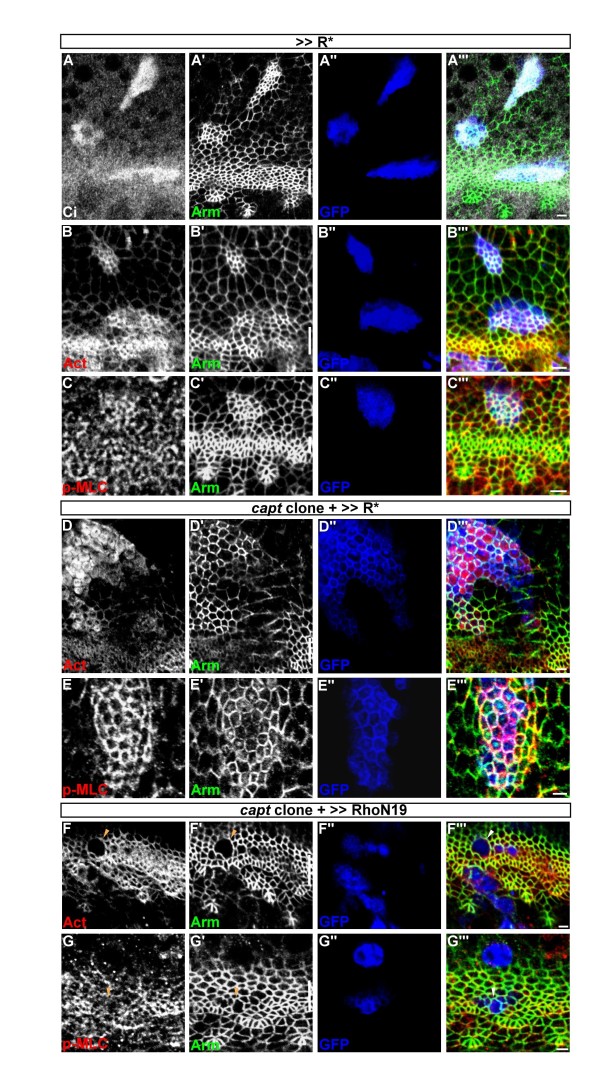
***capt*mutant phenotype depends on Hh pathway.** (A-C) Flip-out clones overexpressing UAS-R* (GFP, blue) anterior to the MF labeled for Arm (green), Ci155 (white in A), F-actin (red in B) and p-MLC (red in C). (D,E) *capt*^*E636*^ mutant MARCM clones overexpressing UAS-R* (GFP, blue) anterior to the MF labeled for actin (red in D), p-MLC (red in E) and Arm (green). (F,G) *capt*^*E636*^ mutant MARCM clones (GFP, blue) overexpressing *UAS-Rho*^*N19*^ labeled for actin (red in F), p-MLC (red in G) and Arm (green). Arrowheads indicate *capt*^*E636*^ mutant cells. In all images, lines indicate the position of the MF, and anterior is to the top. Bars, 2 μm.

Hh signaling is required to promote apical constriction downstream of Ci in the MF [9,14]. We showed that the effects of *capt/ssh* in F-actin/p-MLC accumulation were only confined to cells within and posterior to the MF (Figures 2C3C). As these cells all received Hh signal beforehand, we next determined whether the described *capt* phenotype is dependent on Hh-mediated apical constriction. Protein kinase A (PKA) phosphorylates Ci155 to promote its proteolysis into the Ci75 repressor in cells anterior to the MF and this proteolysis is blocked by the activation of Hh signaling within the MF. We first generated ectopic clones, anterior to the MF, overexpressing the regulatory subunit of PKA (R*) to sequester the catalytic subunit (Figure 4A) and, therefore, to activate Hh signalling by upregulating the levels of Ci155 [26]. As expected, we observed ectopic apical constriction in cells anterior to the MF, with enrichment of F-actin and p-MLC to a level similar to cells within the MF (Figure 4B, C). We then generated *capt*^*E636*^ mutant clones anterior to the MF using MARCM overexpressing R*. Interestingly, similar to *capt*^*E636*^ mutant clones within and posterior to the MF, much stronger F-actin (Figure 4D) and p-MLC (Figure 4E) accumulation as well as discontinuous AJs (Figure 4D′, E′) were detected in *capt*^*E636*^ mutant clones anterior to the MF. These data indicate that the *capt* mutant phenotype anterior to the MF is dependent on the inactivation of PKA activity or the resultant accumulation of Ci155/removal of Ci75.

It was shown that Hh signaling acts through Ci to regulate Rho/Rok/myosin II to cause apical constriction [9]. We next determined whether the accumulation of p-MLC and F-actin in *capt* mutant cells is dependent on the activation of the downstream effector Rho. To test this, we overexpressed a dominant negative form of Rho (Rho^N19^) in *capt*^*E636*^ mutant clones using MARCM. These clones tended to be small, however, we found that inhibition of Rho activity abolished *capt*-mediated accumulation of F-actin and p-MLC within and posterior to the MF (Figure 4F,G), indicating that the accumulation of p-MLC and F-actin in *capt* mutant cells required the activation of Rho. Together, our results suggest that the *capt* mutant phenotypes detected were dependent on the inactivation of PKA and activation of Rho.

## Discussion

### Phenotypic differences between *Capt/ssh* and *tsr*

Cofilin is activated by Ssh, the phenotypic differences between *capt/ssh* and *tsr* mutant MF cells are unexpected. However, as mentioned in the Introduction, *RNAi* depletion of *tsr* and *ssh* in *Drosophila* S2 cells affects actin-based lamella formation differently [10]. Recent studies also show that loss of *capt*, but not *tsr*, leads to activation and nuclear import of Yorkie in the wing disc [27,28]. Thus, the effect generated by the lack of cofilin (as in *tsr* mutant cells) is not completely the same as disrupting cofilin and p-cofilin recycling (as in *ssh* mutant cells). The reason for this is unclear. One possibility is that cofilin activity is regulated by multiple additional mechanisms, including dephosphorylation of phosphorylated cofilin by chronophin and general phosphatase [29]. Alternatively, Capt and Ssh have a common function independent of cofilin.

### Capt/ssh affects AJs

Spot AJs in early embryonic epithelia contain homophilic DE-cad clusters in a stable microdomain and are bona fide sites of adhesion [30]. Small but stable actin patches were suggested to underlie the stability of spot AJs [30]. We found that, in actin-turnover defective *capt/ssh* mutant cells, fragmented AJs were stable and they assembled at regions where cadherin and high levels of F-actin overlapped. Thus, fragmented AJs and spot AJs might have a similar function but they differ in underlying F-actin organization. We showed that the fragmented AJs can also non-autonomously assemble along the interface between wild-type cells when they were surrounded by the *capt*^*E636*^ mutant cells. One possibility is that when there is a fragmented AJ formed at one side of a wild-type cell (along the interface between wild-type and *capt*^*E636*^ mutant cells), this fragmented AJ cannot link to the adjacent AJ (along the interface between wild-type cells) at the tricellular junction, and therefore this adjacent AJ also becomes fragmented once the associated actomyosin contracts.

### Capt/ssh and premature photoreceptor differentiation

It has been proposed that *capt* is required to prevent premature photoreceptor differentiation ahead of the MF [7]. Here, we showed that the *capt/ssh* mutant phenotypes, including the apical surface area, cannot be detected anterior to the MF. By using the enrichment of EGF receptor to mark MF cells [31], we observed roughly 8–10 rows of enlarged *capt* mutant cells with enrichment of EGF receptor in a *capt* mutant clone spanning the MF (Additional file 1: Figure S3A). This is similar to the presence of about 10 rows of endogenous MF cells in the wild-type disc [9]. Thus, the numbers of MF cells are largely the same in both wild-type and *capt* mutant discs. However, as the *capt* mutant MF cells have enlarged apical area, they might occupy positions anterior to the endogenous MF and thus cause the distortion of the MF toward the anterior of the MF (Additional file 1: Figure S3A). However, this distortion of the MF, at lower resolution, was previously interpreted as premature photoreceptor differentiation caused by anteriorly diffused Hh [7].

## Conclusions

It was previously shown at low resolution that *capt, ssh* and *tsr* mutant cells all similarly cause accumulation of F-actin and enlarged apical area in the eye epithelia [7],[9]. Here, we found that *capt* and *ssh*, but not *tsr*, mutant cells share various phenotypes that are dependent on the inactivation of PKA and activation of Rho. In addition to the accumulation of F-actin as previously reported, these phenotypes also exhibited excessive accumulation of p-MLC, fragmented AJs and hexagonal cell shape.

## Competing interests

The authors declare that they have no competing interests.

## Authors’ contribution

CML designed and performed experiments. JCH designed experiments and wrote the manuscript. PYL and YCL helped the genetic crosses and analyzed the data. All authors read and approved the final manuscript.

## Supplementary Material

Additional file 1 **Figure S1.** Apical cell surface. The paired Student’s *t*-test was applied. **Figure S2.** Percentage of cells with x-sided polygons. The paired Student’s *t*-test was applied. **Figure S3.***captE636* mutant MARCM clones labeled for Arm (green), EGFR (red) and GFP (blue). Bracket indicates 8-10 rows of *captE636* mutant MF cells (dots) with elevated levels of EGFR. Bars, 2 μm. Click here for file

## References

[B1] Gieselmann R Mann K ASP-56, a new actin sequestering protein from pig platelets with homology to CAP, an adenylate cyclase-associated protein from yeast FEBS Lett 1992 298 149 153 10.1016/0014-5793(92)80043-G 1544438

[B2] Zelicof A Protopopov V David D Lin XY Lustgarten V Gerst JE Two separate functions are encoded by the carboxyl-terminal domains of the yeast cyclase-associated protein and its mammalian homologs. Dimerization and actin binding J Biol Chem 1996 271 18243 18252 10.1074/jbc.271.30.18243 8663401

[B3] Lappalainen P Drubin DG Cofilin promotes rapid actin filament turnover in vivo Nature 1997 388 78 82 10.1038/40418 9214506

[B4] Arber S Barbayannis FA Hanser H Schneider C Stanyon CA Bernard O Caroni P Regulation of actin dynamics through phosphorylation of cofilin by LIM-kinase Nature 1998 393 805 809 10.1038/31729 9655397

[B5] Niwa R Nagata-Ohashi K Takeichi M Mizuno K Uemura T Control of actin reorganization by Slingshot, a family of phosphatases that dephosphorylate ADF/cofilin Cell 2002 108 233 246 10.1016/S0092-8674(01)00638-9 11832213

[B6] Baum B Li W Perrimon N A cyclase-associated protein regulates actin and cell polarity during Drosophila oogenesis and in yeast Curr Biol 2000 10 964 973 10.1016/S0960-9822(00)00640-0 10985383

[B7] Benlali A Draskovic I Hazelett DJ Treisman JE act up controls actin polymerization to alter cell shape and restrict Hedgehog signaling in the Drosophila eye disc Cell 2000 101 271 281 10.1016/S0092-8674(00)80837-5 10847682

[B8] Chen J Godt D Gunsalus K Kiss I Goldberg M Laski FA Cofilin/ADF is required for cell motility during Drosophila ovary development and oogenesis Nat Cell Biol 2001 3 204 209 10.1038/35055120 11175754

[B9] Corrigall D Walther RF Rodriguez L Fichelson P Pichaud F Hedgehog signaling is a principal inducer of Myosin-II-driven cell ingression in Drosophila epithelia Dev Cell 2007 13 730 742 10.1016/j.devcel.2007.09.015 17981140

[B10] Rogers SL Wiedemann U Stuurman N Vale RD Molecular requirements for actin-based lamella formation in Drosophila S2 cells J Cell Biol 2003 162 1079 1088 10.1083/jcb.200303023 12975351 PMC2172842

[B11] Heberlein U Wolff T Rubin GM The TGF beta homolog dpp and the segment polarity gene hedgehog are required for propagation of a morphogenetic wave in the Drosophila retina Cell 1993 75 913 926 10.1016/0092-8674(93)90535-X 8252627

[B12] Ma C Zhou Y Beachy PA Moses K The segment polarity gene hedgehog is required for progression of the morphogenetic furrow in the developing Drosophila eye Cell 1993 75 927 938 10.1016/0092-8674(93)90536-Y 8252628

[B13] Dominguez M Hafen E Hedgehog directly controls initiation and propagation of retinal differentiation in the Drosophila eye Genes Dev 1997 11 3254 3264 10.1101/gad.11.23.3254 9389656 PMC316756

[B14] Escudero LM Bischoff M Freeman M Myosin II regulates complex cellular arrangement and epithelial architecture in Drosophila Dev Cell 2007 13 717 729 10.1016/j.devcel.2007.09.002 17981139

[B15] Nagata-Ohashi K Ohta Y Goto K Chiba S Mori R Nishita M Ohashi K Kousaka K Iwamatsu A Niwa R A pathway of neuregulin-induced activation of cofilin-phosphatase Slingshot and cofilin in lamellipodia J Cell Biol 2004 165 465 471 10.1083/jcb.200401136 15159416 PMC2172350

[B16] Li W Ohlmeyer JT Lane ME Kalderon D Function of protein kinase A in hedgehog signal transduction and Drosophila imaginal disc development Cell 1995 80 553 562 10.1016/0092-8674(95)90509-X 7867063

[B17] Wang D Zhang L Zhao G Wahlstrom G Heino TI Chen J Zhang YQ Drosophila twinfilin is required for cell migration and synaptic endocytosis J Cell Sci 2010 123 1546 1556 10.1242/jcs.060251 20410372

[B18] Strutt DI Weber U Mlodzik M The role of RhoA in tissue polarity and Frizzled signalling Nature 1997 387 292 295 10.1038/387292a0 9153394

[B19] Oda H Tsukita S Real-time imaging of cell-cell adherens junctions reveals that Drosophila mesoderm invagination begins with two phases of apical constriction of cells J Cell Sci 2001 114 493 501 11171319 10.1242/jcs.114.3.493

[B20] Lee T Luo L Mosaic analysis with a repressible cell marker for studies of gene function in neuronal morphogenesis Neuron 1999 22 451 461 10.1016/S0896-6273(00)80701-1 10197526

[B21] Stowers RS Schwarz TL A genetic method for generating Drosophila eyes composed exclusively of mitotic clones of a single genotype Genetics 1999 152 1631 1639 10430588 10.1093/genetics/152.4.1631 PMC1460682

[B22] Ito K Awano W Suzuki K Hiromi Y Yamamoto D The Drosophila mushroom body is a quadruple structure of clonal units each of which contains a virtually identical set of neurones and glial cells Development 1997 124 761 771 9043058 10.1242/dev.124.4.761

[B23] Chang WL Liou W Pen HC Chou HY Chang YW Li WH Chiang W Pai LM The gradient of Gurken, a long-range morphogen, is directly regulated by Cbl-mediated endocytosis Development 2008 135 1923 1933 10.1242/dev.017103 18434418

[B24] Martin AC Pulsation and stabilization: Contractile forces that underlie morphogenesis Dev Biol 2010 341 114 125 10.1016/j.ydbio.2009.10.031 19874815

[B25] Goode BL Drubin DG Lappalainen P Regulation of the cortical actin cytoskeleton in budding yeast by twinfilin, a ubiquitous actin monomer-sequestering protein J Cell Biol 1998 142 723 733 10.1083/jcb.142.3.723 9700161 PMC2148182

[B26] Ou CY Lin YF Chen YJ Chien CT Distinct protein degradation mechanisms mediated by Cul1 and Cul3 controlling Ci stability in Drosophila eye development Genes Dev 2002 16 2403 2414 10.1101/gad.1011402 12231629 PMC187440

[B27] Fernandez BG Gaspar P Bras-Pereira C Jezowska B Rebelo SR Janody F Actin-Capping Protein and the Hippo pathway regulate F-actin and tissue growth in Drosophila Development 2011 138 2337 2346 10.1242/dev.063545 21525075

[B28] Sansores-Garcia L Bossuyt W Wada K Yonemura S Tao C Sasaki H Halder G Modulating F-actin organization induces organ growth by affecting the Hippo pathway EMBO J 2010 30 2325 2335 21556047 10.1038/emboj.2011.157 PMC3116287

[B29] Oser M Condeelis J The cofilin activity cycle in lamellipodia and invadopodia J Cell Biochem 2009 108 1252 1262 10.1002/jcb.22372 19862699 PMC3718474

[B30] Cavey M Rauzi M Lenne PF Lecuit T A two-tiered mechanism for stabilization and immobilization of E-cadherin Nature 2008 453 751 756 10.1038/nature06953 18480755

[B31] Ho YH Lien MT Lin CM Wei SY Chang LH Hsu JC Echinoid regulates Flamingo endocytosis to control ommatidial rotation in the Drosophila eye Development 2010 137 745 754 10.1242/dev.040238 20110316

