# Supplementary material for: Capulet and Slingshot share overlapping functions during Drosophila eye morphogenesis
Source: J Biomed Sci. 2012 Apr 30;19(1):46. doi: 10.1186/1423-0127-19-46 (PMC3411472; doi:10.1186/1423-0127-19-46)
Supplement: Additional file 1 — Figure S1. Apical cell surface. The paired Student’s t-test was applied. Figure S2. Percentage of cells with x-sided polygons. The paired Student’s t-test was applied. Figure S3.captE636 mutant MARCM clones labeled for Arm (green), EGFR (red) and GFP (blue). Bracket indicates 8-10 rows of captE636 mutant MF cells (dots) with elevated levels of EGFR. Bars, 2 μm. [file 1423-0127-19-46-S1.doc]

**Online supplemental material**

**
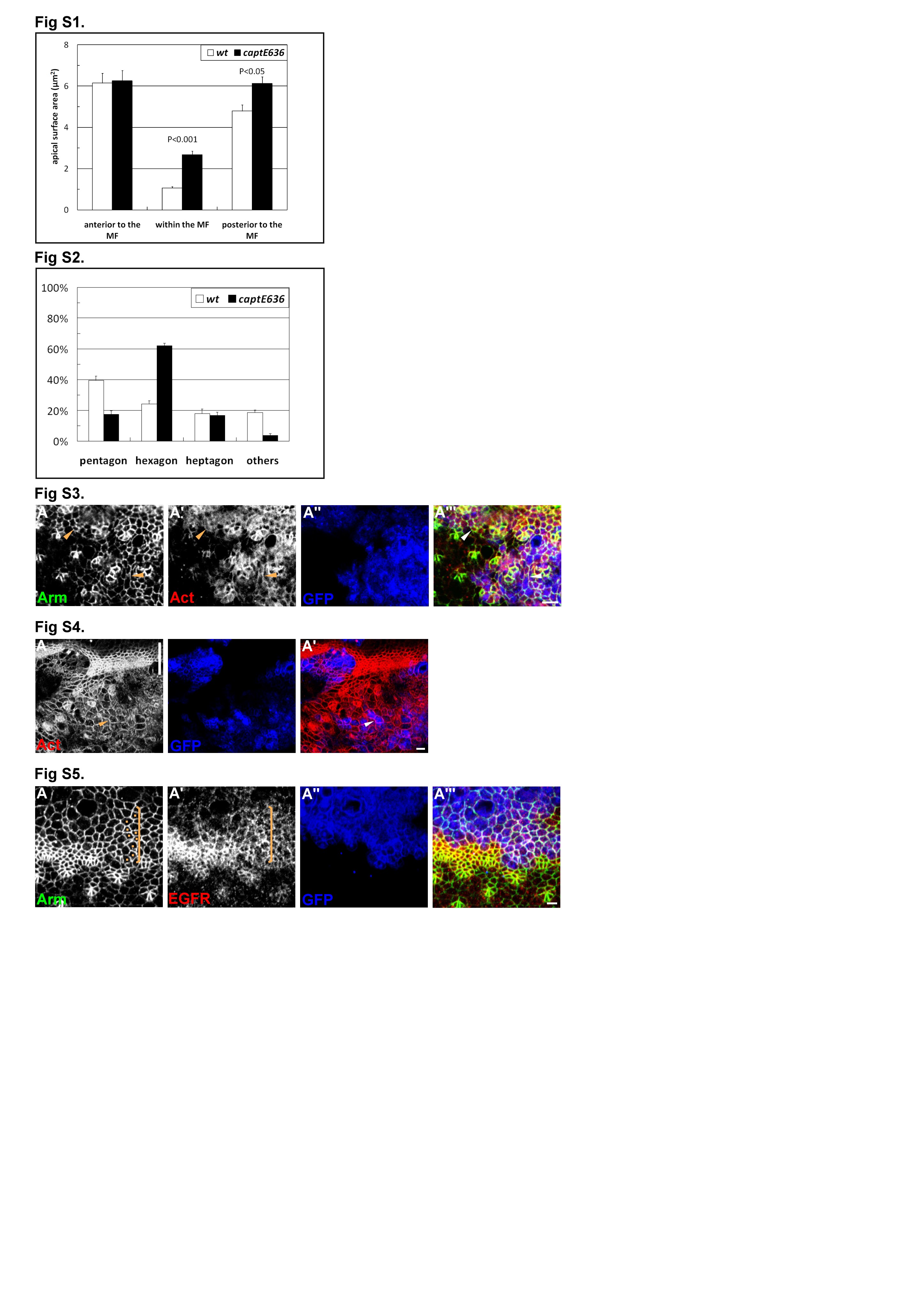
**

### Supplementary Figure 1. Apical cell surface. The paired Student’s *t*-test was applied.


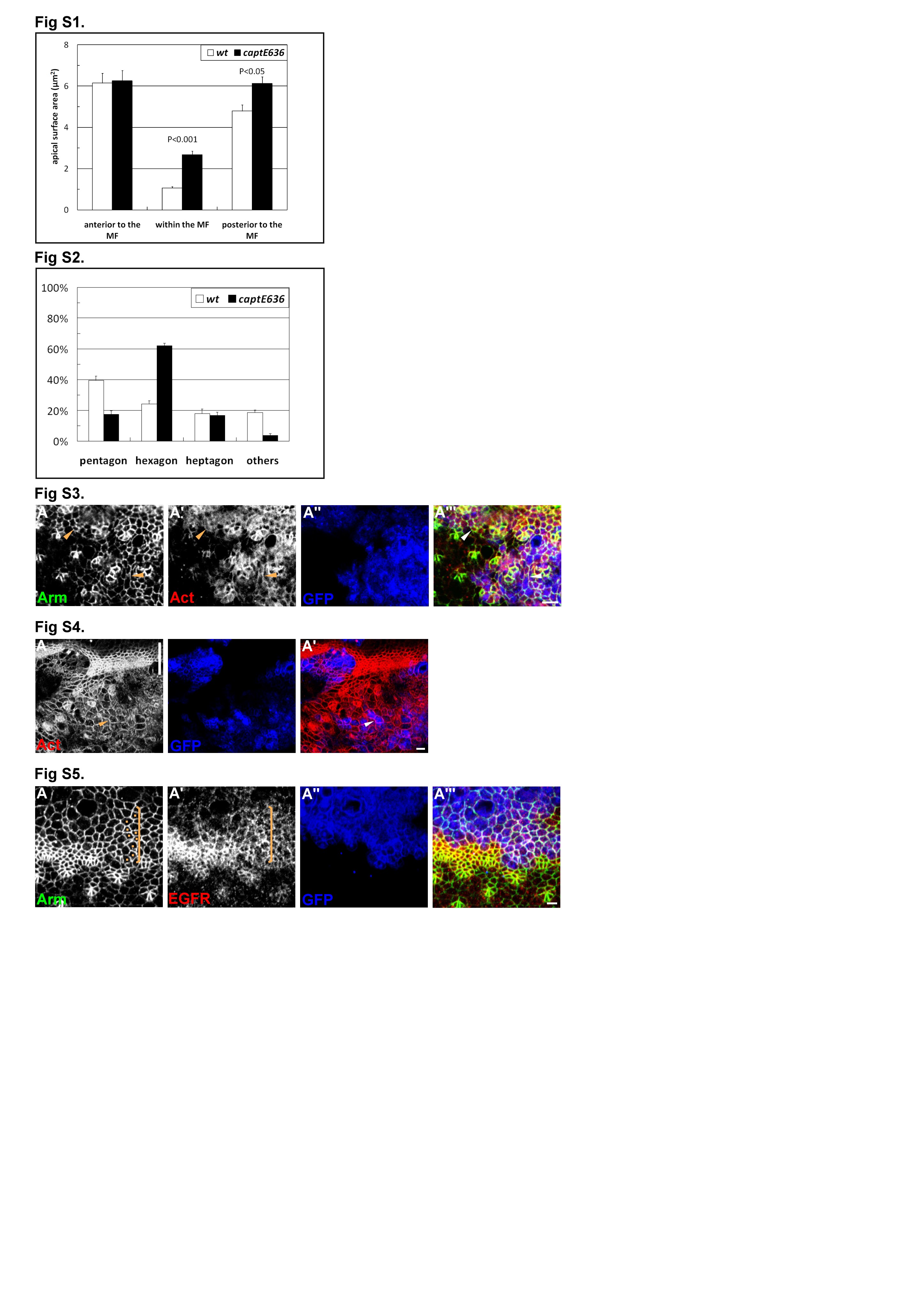


### Supplementary Figure 2. Percentage of cells with x-sided polygons. The paired Student’s *t*-test was applied.


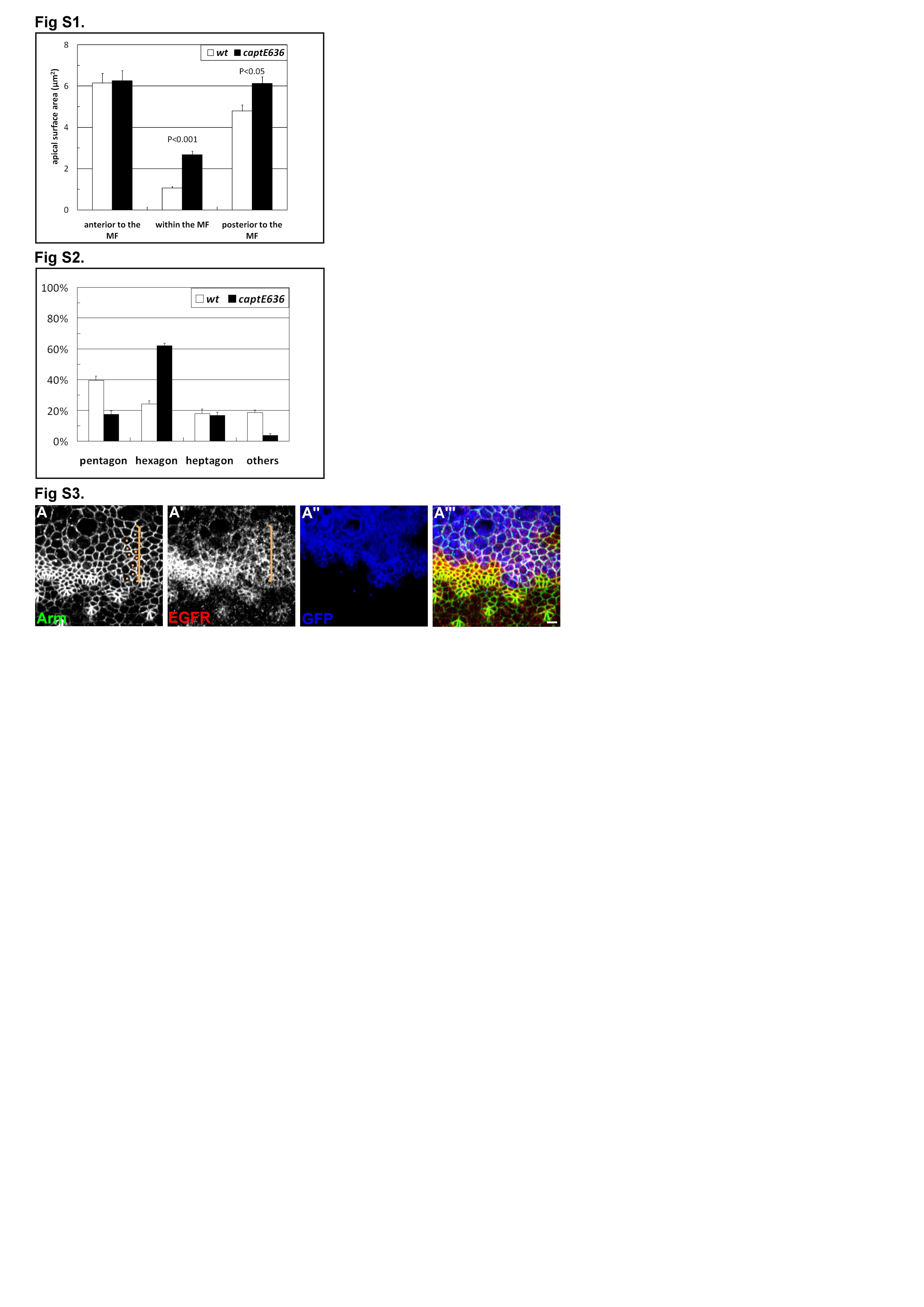


**Supplementary Figure 3.** *captE636* mutant MARCM clones labeled for Arm (green), EGFR (red) and GFP (blue). Bracket indicates 8-10 rows of *captE636* mutant MF cells (dots) with elevated levels of EGFR. Bars, 2 μm.
